# Opportunities and Challenges for Successful Use of Bevacizumab in Pediatrics

**DOI:** 10.3389/fonc.2013.00092

**Published:** 2013-04-29

**Authors:** Amy Barone, Joshua B. Rubin

**Affiliations:** ^1^Division of Pediatric Hematology and Oncology, Department of Pediatrics, St. Louis Children’s Hospital, Washington University School of MedicineSt. Louis, MO, USA

**Keywords:** anti-angiogenic, bevacizumab, VEGF, pediatric, glioblastoma

## Abstract

Bevacizumab (Avastin) has rapidly gained status as a broadly active agent for malignancies of several different histologies in adults. This activity has spawned a range of uses in pediatrics for both oncologic and non-oncologic indications. Early analyses indicate that pediatric cancers exhibit a spectrum of responses to bevacizumab that suggest its activity may be more limited than in adult oncology. Most exciting, is that for low-grade tumors that threaten vision and hearing, there is not only evidence for objective tumor response but for recovery of lost function as well. In addition to oncological indications, there is a range of uses for non-oncologic disease for which bevacizumab has clear activity. Finally, a number of mechanisms have been identified as contributing to bevacizumab resistance in cancer. Elucidating these mechanisms will guide the development of future clinical trials of bevacizumab in pediatric oncology.

## Introduction

Angiogenesis, or the formation of new blood vessels from pre-existing vessels is essential for normal organogenesis and tumorigenesis. The wide range of requirements for new blood vessel growth during development and in the setting of pathology is served by numerous angiogenic factors that recruit endothelial progenitors and other pro-angiogenic and vascular supporting cells, and promote endothelial cell proliferation and survival. Vascular endothelial growth factor (VEGF) is essential to both normal and pathology-associated angiogenesis and consequently, VEGF antagonism is the primary focus of anti-angiogenic therapies. Multiple isoforms of VEGF are generated through alternate splicing and post-translational modifications of the products of a multi-gene family comprised of VEGF-A, -B, -C, -D and placenta growth factor (Ferrara, 2004). Most of the VEGF angiogenic activity in normal and pathological conditions can be ascribed to VEGF-A (Leung et al., 1989). VEGF-A (VEGF) expression is regulated by hypoxia (Goldberg and Schneider, 1994) through a HIF-1 dependent mechanism (Ema et al., 1997), as well as by a large number of secreted hormones and cytokines that provide for tissue specific and physiologically appropriate angiogenic responses (Ferrara, 2004). Several primary oncogenic events including, mutational activation of the Ras (Grugel et al., 1995) and Wnt (Zhang et al., 2001) pathways, also upregulate VEGF expression and VEGF is known to be overexpressed in many types of cancer including colon cancer, non-squamous non-small cell lung cancer (NSCLC), kidney cancer, and brain cancers. In brain tumors, VEGF is highly expressed by both tumor cells and tumor-associated vascular endothelial cells (Takahashi et al., 1995; Fidler, 2001; Huber et al., 2001; Salmaggi et al., 2003; Norden et al., 2008). The resultant neovasculature provides circulatory support for tumor growth and is thought to be important as a tumor stem cell niche and a conduit for tumor invasion and metastasis (Takahashi et al., 1995; Fidler, 2001). Thus, molecular inhibition of VEGF is an attractive model for targeting tumor growth and spread.

Biological activity of VEGF is further regulated through the generation of alternate splice forms, their post-translational modification and their sequestration, and release from cell surface and extracellular matrix heparin sulfate binding sites. Four primary alternately spliced isoforms of VEGF are produced (VEGF_121_, VEGF_165_, VEGF_189_, VEGF_206_) (Houck et al., 1992). These differ in their affinities for heparin sulfate proteoglycans (HSPGs) and thus the extent to which they are freely diffusible. VEGF_121_ does not bind HSPGs while VEGF_189_ and VEGF_206_ are restricted in their diffusion by high affinity interactions with HSPGs. VEGF_165_ exhibits intermediate affinity and diffusibility. Genetic studies indicate that VEGF-HSPG interactions are required for establishing VEGF gradients and normal angiogenesis (Carmeliet et al., 1996; Ruhrberg et al., 2002). Proteolytic processing of VEGF by plasmin (Keyt et al., 1996) and MMPs (Lee et al., 2005) further regulates its interactions with HSPG and biological activity.

Vascular endothelial growth factor binds two receptor tyrosine kinase VEGFR-1 and VEGFR-2 (KDR) as well as two co-receptor, Neurophilin 1 and 2. While binding to VEGFR-1 occurs with higher affinity than binding to VEGFR-2, it is VEGFR-2 that mediates the pro-angiogenic effects of VEGF on endothelial cells (Olsson et al., 2006). Thus, antagonism of VEGF can be achieved by blocking its bioavailability or by inhibiting its actions through VEGFR-2.

In 2004, bevacizumab, a monoclonal antibody directed at all five isoforms of VEGF, was FDA approved to treat NSCLC and has since been approved to treat metastatic colorectal cancer, metastatic kidney cancer, and glioblastoma (GBM) in patients who had progressed after prior treatment (Cohen et al., 2007a,b, 2009). It was the first drug developed to exclusively inhibit angiogenesis. Encouraging data from adult studies has led to an increased use of bevacizumab in the pediatric population (Bokstein et al., 2008; Buie and Valgus, 2008; Narayana et al., 2009). In this article, we will review the current clinical applications of bevacizumab in pediatrics and proposed mechanisms of resistance.

## Brain Tumors

### High-grade gliomas

Outcomes for children with high-grade gliomas (HGG) remain dismal with 5-year-survival rates of 5–15% for grade IV gliomas and 20–40% for grade III gliomas (Mueller and Chang, 2009; Narayana et al., 2010). HGG characteristically posses high levels of VEGF expression making bevacizumab, a attractive therapeutic option (Oehring et al., 1999; Narayana et al., 2009; Keunen et al., 2011). Several adult studies demonstrated increased progression-free survival (PFS) and overall survival (OS) in patients with GBM treated with bevacizumab (Bokstein et al., 2008; Buie and Valgus, 2008; Kreisl et al., 2009; Narayana et al., 2009). This encouraging data stimulated VEGF use in pediatric patients. Multiple initial reports described bevacizumab (10 mg/kg every other week) with and without irinotecan (125 mg/kg every other week) in small series of 3–12 patients with mixed diagnoses of recurrent or refractory tumors. In all cases, initial responses were evident in a subset of patients with variable impact on survival (Carmeliet et al., 1996; Keyt et al., 1996; Ruhrberg et al., 2002) (see Table 1). The largest retrospective review by Couec et al. (2012) involved 28 pediatric patients with recurrent or refractory brain tumors treated with bevacizumab and irinotecan. Ten out of 12 patients with HGG had a median PFS of 8 weeks and the authors concluded that more prospective trials would be necessary to determine efficacy in patients with HGGs (Couec et al., 2012). In 2010, the only prospective clinical trial results for children with HGG were published by the Pediatric Brain Tumor Consortium (Gururangan et al., 2010). The study included 31 patients, 15 with GBM and 16 with diffuse intrinsic pontine glioma (DIPG); all were treated with bevacizumab (10 mg/kg/dose) and irinotecan (125 mg/m^2^ increased to 150 mg/m^2^ as tolerated or 250 mg/m^2^ increased to 350 mg/m^2^) every 2 weeks. Fifteen of the patients had no objective sustained response. Eight of the patients with GBM had stable disease for ≥12 weeks with a median of 176 days and median PFS of 4.2 months. Median PFS for patients with DIPG was 2.3 months. This study concluded that there is a lack of efficacy of combined bevacizumab and irinotecan in children with recurrent GBM and DIPG.

**Table 1:** **Efficacy results for high-grade glioma**.

Study	Benesch et al. (2008)	Narayana et al. (2010)	Parekh et al. (2011)	Couec et al. (2012)	Gururangan et al. (2010)
**Patient number**	*n* = 3	*n* = 10 HGG; *n* = 2 DIPG	*n* = 8	*n* = 10 HGG	*n* = 15 HGG; *n* = 16 DIPG
**Treatment**	Bevacizumab ± other agent	Bevacizumab and irinotecan	Bevacizumab ± other agent	Bevacizumab and irinotecan	Bevacizumab and irinotecan
**Imaging**	Initial tumor SD or PR but eventual progression	No CR	Contrast enhancing lesions – PR or SD in five of seven patients	10 of 12 PD (median 8 weeks post start of treatment)	No SD
		25% had PR on MRI perfusion 2 SD	Non-contrast enhancing lesions – PD in three of four patients		
**Median PFS and OS**		PFS 2.25 months	PFS 15 weeks	PFS 8 weeks	PFS 4.2 months (HGG)
		OS 6.35 months	OS 30.5 weeks		PFS 2.3 months (DIPG)

### Low-grade gliomas

Low-grade gliomas (LGG) are the most common childhood brain tumor. Treatment varies as a function of age and tumor location between surgical resection alone, or combined surgery and chemo or radiation therapy. Common indications for adjuvant therapy include recurrence in surgically inaccessible locations and threats to function particularly for deep-seated midline tumors growth of which can result in vision loss, or hypothalamic or brainstem dysfunction. While LGG are slow growing, biologically benign tumors, they possess increased vasculature (Plate et al., 1992; Takahashi et al., 1995; Oehring et al., 1999) and thus are also an attractive indication for anti-angiogenic therapy. In 2009, Packer et al. (2009) conducted a retrospective review of 10 pediatric patients with recurrent LGG, 3 with NF1, treated with bevacizumab (10 mg/kg/dose) and irinotecan (125 mg/m^2^/dose) every 2 weeks. Seven had a radiographic response (1 CR, 3 PR, and 3 minor response) and seven had clinical improvement [improvement in visual acuity (*n* = 2), improved hemiparesis (*n* = 2), weight gain with improved diencephalic syndrome (*n* = 4), decreased seizure activity (*n* = 2), and reversal of psychomotor retardation (*n* = 2)]. At the conclusion of the study, eight of the nine patients had not progressed (see Table 2). In 2012, Hwang et al. (2013) extended the Packer et al. report with a retrospective review of 14 children (10 of whom were included in the study by Packer et al.) with multiple recurrent LGGs treated with bevacizumab-based therapy for at least 12 months, at least 1 year out from completion of therapy. Most were treated with bevacizumab 10 mg/kg and irinotecan 125 mg/m^2^ every 2 weeks; one was treated with bevacizumab alone every 3 weeks. After two cycles, 12 of 14 patients had an objective response and two had stable disease. Metastatic disease responses generally correlated with primary tumor response. Four patients had improved vision, two had improved ataxia, one had improved hemiparesis, four had weight gain with diencephalic syndrome, and three had improved developmental milestones. No difference in response was seen between grade I and grade II tumors. Four patients were retreated with bevacizumab when they progressed clinically or radiographically after the discontinuation of bevacizumab. All had clinical or radiographic improvement while on therapy, however, all progressed when bevacizumab was discontinued. Two received a third treatment course; one of these patients had NF1 and had significant improvement of vision with every treatment course that would worsen each time it was stopped. Progression occurred after discontinuation of therapy in 13 of 14 (range 4–16 months). The 14th patient was progression-free at 45 months. Significant responses to bevacizumab in children with LGG is consistent with reported activity for other anti-angiogenic therapies such as thalidomide-based regimens (Short et al., 2001; Gilheeney et al., 2007) or weekly vinblastine (Lafay-Cousin et al., 2005) and suggest that anti-angiogenic therapy is an effective approach to treatment of any patient with LGG requiring adjuvant therapy. Particularly exciting is the observed improvement in vision with bevacizumab treatment.

**Table 2:** **Efficacy results for low-grade glioma, NF2, and other CNS malignancies**.

Disease	LGG	LGG	LGG	Schwannoma with NF2	Schwannoma with NF2	Ependympoma	Medulloblastoma
**Study**	Packer et al. (2009)	Hwang et al. (2013) (includes data from Packer et al., 2009)	Couec et al. (2012)	Plotkin et al. (2009)	Mautner et al. (2010)	Gururangan et al. (2012)	Aguilera et al. (2011)
**Number**	*n* = 10	*n* = 14	*N* = 7	*N* = 10	*N* = 2	*N* = 15	*N* = 2
**Treatment**	Bevacizumab and irinotecan	Bevacizumab ± other	Bevacizumab and irinotecan	Bevacizumab	Bevacizumab	Bevacizumab and irinotecan	Bevacizumab and irinotecan or temozolomide
**Imaging**	1 CR; 3 PR; 3 minor response	12 PR; 2 SD	6 of 7 PR	6 PR; 3 SD	Decreased tumor size and resolution of brainstem compression.	No objective response; 2 SD	CR in both patients
**Clinical Outcome**	Improved visual acuity (*n* = 2), improved hemiparesis (*n* = 2), weight gain with improved diencephalic syndrome (*n* = 4), decreased seizure activity (*n* = 2), and reversal of psychomotor retardation (*n* = 2)	Improved vision (*n* = 4), improved ataxia (*n* = 2), improved hemiparesis (*n* = 1)	Data not available	Improved hearing and word recognition (*n* = 4). Improved headaches and vomiting (*n* = 1). Decline in word recognition despite tumor shrinkage (*n* = 1). Stable hearing despite tumor shrinkage (*n* = 1)	First patient: slight improvement in hearing and word recognition. Improved ability to communicate over the phone and distinguish voices.	Data not available	Excellent quality-of-life
					Second patient: no improvement in clinical hearing per patient self-report		
**Median PFS and OS**	Eight of nine had not progressed at conclusion of study	None progressed on treatment. Time to progression after stopping treatment ranged from 4 to 16 months (*n* = 13). 14th patient was progression-free at 45 months	All alive 1 of 6 PD	All alive	All alive	PD in 10 patients (median time 2.2 months). 6-month PFS overall 25.7%	First patient: stable at 35 months; therapy discontinued after 24 months. Second patient: malignant cells in CSF after 18 months

### Neurofibromatosis type 2

Bilateral vestibular Schwannomas are a frequent and highly morbid complication of Neurofibromatosis type 2 (NF2). Although benign, these tumors tend to inexorably progress and often result in loss of all functional hearing by middle age. Standard treatment includes surgery and radiation however, these approaches are frequently ineffective at preserving hearing (Briggs et al., 1994; Mautner et al., 2010). Schwannomas express VEGF and the VEGF-1 receptor; and higher levels are associated with increased rates of tumor growth (Plotkin et al., 2009).

In 2009, Plotkin et al. conducted a retrospective study of 10 patients with NF2 (ages 16–53) treated with bevacizumab (5 mg/kg every 2 weeks for 12 months) (Di Tomaso et al., 2011). On imaging, 6 of the 10 had partial response and 3 has stable disease (median response 26% reduction, range 44% shrinkage to 32% growth). Of the six, four remained stable 11–16 months later (see Table 2). There was a strong correlation between mean apparent diffusion coefficient (ADC) at baseline within the tumor and percent decrease in volume at 3 months (*p* = 0.001). Importantly, four of the seven evaluable patients had 57% improvement in word recognition beginning approximately 8 weeks after initiation of therapy and four of those five also reported improvement in tinnitus. Mautner et al. (2010) reported two cases where bevacizumab had similar benefits for patients with NF2 and vestibular Schwannomas (see Table 2). Similar to the experience in optic pathway gliomas, these studies are particularly compelling for the recovery of function observed with bevacizumab treatment.

### Other CNS tumors

Ependymomas are also associated with VEGF expression and the level of VEGF is correlated with prognosis providing a rationale for bevacizumab treatment (Korshunov et al., 2012). In 2012, the Pediatric Brain Tumor Consortium reviewed the use of bevacizumab and irinotecan in 15 patients with recurrent ependymoma (one unevaluable) (Gururangan et al., 2012). No patient had a sustained objective response (see Table 2). Two patients had stable disease (12 and 10 months) and an additional patient had stable disease at 4 months but was removed from study. The study concluded that combination of bevacizumab and irinotecan was not efficacious for the treatment of ependymoma. This is in contrast to reports of more durable responses to a thalidomide-based anti-angiogenic regimen for relapsed ependymoma (Gilheeney et al., 2007).

Two patients with recurrent medulloblastoma treated with bevacizumab and irinotecan or temozolomide were described in a report by Aguilera et al. (2011) (see Table 2). The first patient was a 7-year-old with anaplastic medulloblastoma with a spinal lesion who had a persistent spinal lesion upon completion of therapy. After 20 months of treatment with bevacizumab, irinotecan, and temozolomide, her brain MRI showed a complete response and her spine MRI was stable. She remains stable at 35 months. The second patient was a 5-year-old also with metastatic medulloblastoma who developed new leptomeningeal disease 6 months post-completion of therapy. He was treated with bevacizumab and irinotecan and 6 months after starting treatment MRI showed complete response. Unfortunately, 12 months later, he had malignant cells in the CSF.

Together these studies indicate that the use of bevacizumab remains a potential option for pediatric brain tumors of multiple histologies but requires more complete evaluation in prospective clinical trials.

## Non-CNS Malignancies

Given the potential effectiveness in solid tumors, there have been a few pediatric retrospective studies looking at the use of bevacizumab in non-CNS solid tumor malignancies. In 2008, Benesch et al. (2008) conducted a retrospective study in compassionate used of bevacizumab in pediatric patients (See Table 3). Three patients with carcinomas had no stabilization of disease. Two patients with Stage IV neuroblastoma were treated with therapy that included bevacizumab after progression on initial therapy. In one patient, treatment with bevacizumab, carboplatin, and irinotecan resulted in sufficient regression of lesions that complete resection was possible and the patient is in complete remission. The second patient had bone marrow relapse 13 years after diagnosis. He was treated with bevacizumab but died prior to evaluation.

**Table 3:** **Efficacy results for non-CNS malignancies**.

Study	Benesch et al. (2008)					Conde et al. (2011)	Joshi and Banerjee (2008)
**Tumor Type**	Carcinoma (*n* = 3)	Stage IV neuroblastoma (*n* = 2)	Nephroblastoma (*n* = 2)	Sarcomas (*n* = 3)	C4 spinal cord lesion with von Hippel–Lindau (*n* = 1)	Alveolar soft-part sarcoma of tongue (*n* = 1)	Papillary renal cell carcinoma (*n* = 1)
**Treatment**		Bevacizumab, carboplatin and irinotecan	Bevacizumab and topotecan	Bevacizumab and topotecan	Bevacizumab and interferon-alpha2a	Bevacizumab and celexocib as pre-operative therapy	Bevacizumab
**Outcome**	No SD	Patient 1: CR-lesions decreased and were resected. Patient 2: died prior to evaluation	Patient 1: stable lung metastases for 7 months, then progressed. Patient 2: SD for 6 months, then developed ascites and treatment was stopped to avoid thromboembolic events	Alveolar rhabdomyosarcoma: progressed after 6 weeks; pleomorphic Rhabdomyosarcoma: PR but committed suicide after 9 months; parathyroid synovial sarcoma: progressed after 4 months	Treated for 12 months and currently stable	Tumor size decreased by 30%, able to resect. No reoccurrence 3 years later	After 18 months of treatment, no increased update on FDG-PET. No reoccurrence at 8 months after surgery

Of the two patients with nephroblastoma, one had stable lung metastases for 7 months after bevacizumab treatment, and then progressed. The second patient had stabilized abdominal and mediastinal metastases for 6 months after treatment with bevacizumab and topotecan, but then developed refractory malignant ascites and treatment was stopped to avoid thromboembolic events (Benesch et al., 2008).

Three patients with sarcomas were treated with bevacizumab and topotecan. Two of these progressed shortly after starting treatment, one with multifocal relapse of alveolar rhabdomyosarcoma and another with parathyroidal synovial sarcoma. A patient with pleomorphic rhabdomyosarcoma had a partial response, but sadly, committed suicide 9 months into therapy. One patient with von Hippel–Lindau was treated with bevacizumab and interferon-alpha2a for a C4 spinal cord lesion for 12 months and is currently stable (Benesch et al., 2008).

In 2004, Conde et al. (2011) reported the use of bevacizumab as pre-operative management of alveolar soft-part sarcoma of the tongue. Given the hemorrhagic nature of the tumor, anti-angiogenic therapy was used prior to surgery in attempt to reduce the mass to avoid a tongue amputation. After six cycles, tumor size decreased by 30% and she successfully underwent left hemiglossectomy. Three years later, she has not had reoccurrence. In 2008, bevacizumab was used to treat a 14-year-old female with papillary renal cell carcinoma, locally advanced, unresectable who progressed after 10 months of IL-2 therapy (Joshi and Banerjee, 2008). CT showed a partial response after six treatments (10 mg/kg every 2 weeks). After 18 treatments, she had no increased uptake on FDG-PET; no tumor reoccurrence at 8 months after surgery.

Thus, anecdotal evidence suggests there may be specific diagnoses like neuroblastoma and circumstances, including pre-surgical management where bevacizumab therapy may be a benefit in the treatment of children with cancer.

## Malignancy-Related Uses

### Radiation necrosis

Several studies in adult patients have shown a benefit for bevacizumab treatment in the management of post-cranial radiation edema, which can be a significant cause of morbidity in brain tumor patients (Gonzalez et al., 2007; Wong et al., 2008; Matuschek et al., 2011). In 2007, Gonzalez et al. (2007) conducted a retrospective review of brain tumor patients treated with bevacizumab. All of the patients with radiation necrosis (*n* = 8) had a significant reduction in both MRI fluid-attenuated inversion-recovery (FLAIR) and T1-post-Gd contrast abnormalities. The average daily dexamethasone dose was reduced by 8.6 mg. A retrospective review at Children’s Hospital of Denver evaluated the response to bevacizumab of patients with DIPG (*n* = 4) (Liu et al., 2009). In each case, patients suffered clinical decline post-irradiation associated with MRI studies consistent with edema or necrosis rather than tumor progression. Each had initial responses to Decadron and exhibited further improvement with bevacizumab. The combined adult and pediatric experiences would support the use of bevacizumab for the management of post-irradiation edema especially in circumstances where the toxicities of Decadron therapy need to be minimized.

Bevacizumab may also have a role in the treatment of capillary leak syndrome (CLS), a severe complication of allogeneic stem cell transplantation. Yabe et al. (2010) reported that bevacizumab treatment produced rapid improvement of CLS in a 6-year-old male with Fanconi anemia after mismatched unrelated donor stem cell transplant. Sixty-eight days post transplant, he developed CLS, unresponsive to prednisolone, ulinastatin, and albumin. He was then treated with bevacizumab, 5 mg/kg. On day 1, he had improved urine production, blood pressure, and central venous pressure. On day 2, all symptoms resolved. On day 5, he had marked decrease in pleural effusion on CT. He had complete resolution of CLS by day 20.

Vascular endothelial growth factor is a potent inducer of vascular permeability. Blocking it in patients with severe edema such as radiation necrosis and CLS may benefit some patients. Further studies are needed to further evaluate these findings.

## Non-Malignant Uses

### Angiomatosis

Bevacizumab has been used in several case reports of patients with angiomatosis, a neoplasm-like entity. Smith et al. (2008) reported the use of bevacizumab in an infant with cutaneovisceral angiomatosis with thrombocytopenia (CAT) syndrome, a very rare vascular disorder associated with skin and GI lesions and thrombocytopenia. There is no standard of care, although therapy often includes steroids and several agents with known anti-angiogenic activity including vincristine, interferon-alpha2A, and thalidomide. In this report, a 7-month-old was treated with bevacizumab for persistent life-threatening GI bleeds which resolved after three doses. Kline and Buck reported a similar case in a patient with multifocal lymphangioendotheliomatosis with thrombocytopenia (MLT, also known as CAT syndrome) (Kline and Buck, 2009). This patient presented at 8 weeks of life and had multiple GI bleeds despite vincristine, prednisolone, and thalidomide. At 2 years-of-age, she began bevacizumab treatment and subsequently, has only had one GI bleed.

Bevacizumab has also been reported to benefit a patient with multifocal lymphangiomatosis or Gorham–Stout disease (GSD), presenting as idiopathic osteolysis of the bones. GSD is possibly triggered by occult neoplastic cells that produce large amounts of IL-6 and VEGF. Grunewald et al. (2010) reported a 2-year-old with GSD who continued to have progressive osteolysis with massive pleural effusions despite PEG-interferon-alpha-2b, bisphosphonates, and imatinib. Progression of osteolysis and he had no further pleural effusions resolved with the initiation of bevacizumab. He has been stable for 27 months.

### Other

Childhood ophthalmologic disease such as retinopathy of prematurity (ROP), choroidal neovascularization (CNV), Coat’s disease, and familial exudative vitreoretinopathy can lead to severe vision loss. Vision loss can occur for many reasons, including retinal ischemia and VEGF-induced neovascularization and vascular leakage of fluid, blood, and exudates. Mintz-Hittner et al. (2008) conducted a multicenter prospective, randomized control trial to assess intravitreal bevacizumab in infants with zone I or zone II posterior stage 3+ ROP. One hundred fifty infants were randomized to receive bevacizumab or laser therapy bilaterally. Recurrence was seen in only 6 out of 140 eyes in the bevacizumab group and 32 of the 146 eyes in the laser group (*p* = 0.002). Treatment of ROP with bevacizumab is now the standard of care at many centers. Albini and Murry reported a retrospective, non-comparative, open-label, interventional case series in 35 eyes of 33 patients with diseases such as CVN, Coat’s disease, familial exudative vitreoretinopathy and sickle cell retinopathy who were treated with bevacizumab (Sisk et al., 2010). Significant improvement in visual acuity was seen in patients with excess fluid after one or more injections (*p* < 0.05). One patient with sickle cell retinopathy and central retinal vein occlusion had significant improvement in central macular thickness over 6 months after one injection (811–56 μm). Similar effectiveness was also reported for treatment of CNV after Stevens–Johnson syndrome (Kesarwani et al., 2012).

Maturo and Hartnick (2010) reported the use in three children with severe recurrent respiratory papillomatosis. Bevacizumab was thought to be indicated based on increased VEGF receptor expression in pediatric laryngeal papilloma specimens (Rahbar et al., 2005). All three showed an increased time between surgical interventions and two of the three had improvement on quality-of-life scores.

## Adverse Events

In adult studies, serious adverse events have been reported with the use of bevacizumab including CNS hemorrhage (1–5%), hypertensive crisis (<1%), and blood clots (6–9%) (Scappaticci et al., 2007; Nalluri et al., 2008; Marion, 2013). Such risks have made some pediatric practitioners wary of treating with bevacizumab. Fortunately, these events have rarely been reported in children. Multiple retrospective studies reviewed adverse events reported in pediatric patients treated with bevacizumab and no study reported intracerebral hemorrhage, stroke, or thrombosis (see Table 4) (Glade Bender et al., 2008; Joshi and Banerjee, 2008; Packer et al., 2009; Gururangan et al., 2010; Mautner et al., 2010; Narayana et al., 2010; Reismüller et al., 2010; Di Tomaso et al., 2011; Parekh et al., 2011; Couec et al., 2012; Hwang et al., 2013). In 2008, the Children’s Oncology Group conducted a prospective Phase I trial of bevacizumab in pediatric patients with solid tumors (Glade Bender et al., 2008). In this trial, there were no dose-limiting toxicities, no need to discontinue therapy, and no reports of hemorrhage or thrombosis. The most common adverse events reported were Grade I and Grade II events including elevated blood pressure (not typically requiring medical management), proteinuria, lymphopenia, epistaxis, and delayed wound healing. The most commonly reported Grade III events were hypertension (HTN) and proteinuria. Other less common Grade III events included fatigue, poor wound healing, lethargy, transient joint pain, lymphopenia, and pneumonia. Grade IV events were even less common but a few were reported (all *n* = 1) including anaphylaxis, cerebral ischemia, posterior reversible encephalopathic syndrome, proteinuria, lymphopenia, and pneumonia.

**Table 4:** **Adverse events**.

Study	Glade Bender et al. (2008) phase I trial	Parekh et al. (2011)	Narayana et al. (2012a)	Gururangan et al. (2010)	Hwang et al. (2013)	Couec et al. (2012)	Packer et al. (2009)	Reismüller et al. (2010)	Plotkin et al. (2009)	Mautner et al. (2010)	Joshi and Banerjee (2008)
**Tumor type**	Solid tumors	High-grade glioma	Recurrent high-grade glioma	Recurrent malignant glioma and DIPG	Low-grade glioma	Refractory brain tumors	Low-grade glioma	Primary CNS tumors	NF2	NF2	Renal cell carcinoma
**Treatment**	Bevacizumab alone	Combination bevacizumab, irinotecan and temozolomide	Bevacizumab	Bevacizumab and Irinotecan	Bevacizumab-based	Bevacizumab and irinotecan	Bevacizumab and Irinotecan	Bevacizumab	Bevacizumab	Bevacizumab	Bevacizumab
**ADVERSE EVENTS**											
**Grade I/II**	Infusion reaction (*n* = 3), rash (*n* = 3), mucositis (*n* = 2), proteinuria (*n* = 3). Several increases in blood pressure, but no HTN	HTN (*n* = 2), epistaxis (*n* = 2), proteinuria (*n* = 2), local erythema (*n* = 1), defective wound healing (*n* = 1)	Nausea, vomiting, diarrhea, constipation. Skin rash (*n* = 1), intratumoral hemorrhage	Epistaxis (*n* = 1), proteinuria (*n* = 2), fatigue^&^, HTN^&^	Proteinuria (*n* = 3), HTN (*n* = 3), epistaxis (*n* = 2), joint pain (*n* = 1), fatigue (*n* = 3), psychiatric symptoms (*n* = 1)	Hypertension (*n* = 4)***, proteinuria (*n* = 1), lymphopenia (*n* = 2), neutropenia (*n* = 1), thrombocytopenia (*n* = 1), anemia (*n* = 1), elevated transaminases (*n* = 1), electrolyte disturbance (*n* = 1)	Nausea, abdominal pain, obsessive-compulsive behaviors, * increased blood pressure	Proteinuria (*n* = 5), hematuria (*n* = 1), decreased GFR (*n* = 1), lymphopenia (*n* = 7), hypothyroid (*n* = 7), delayed wound healing (*n* = 1)	Elevated AST (*n* = 4), elevated ALT (*n* = 3), proteinuria (*n* = 3), hypertension (*n* = 2), delayed wound healing (*n* = 2), hyperkalemia (*n* = 2), hyperbilirubinemia (*n* = 2), uterine bleeding (*n* = 1), hypocalcemia (*n* = 1), hypophosphatemia (*n* = 1), hypomagnesemia (*n* = 1)	Fatigue, epistaxis, HTN**	Proteinuria after 24 doses, secondary amenorrhea
**Grade III**	–	–	Poor wound healing (*n* = 1) with subsequent DVT (*n* = 1)	Fatigue^&^, HTN^&^, neutropenia (*n* = 1), pancreatitis (*n* = 1)	Lethargy (*n* = 1), proteinuria (*n* = 2), transient joint pain (*n* = 1)		Proteinuria (*n* = 1)	HTN (*n* = 2), proteinuria (*n* = 1), lymphopenia (*n* = 4), pneumonia (*n* = 2			
**Grade IV**	–	–	Anaphylaxis (*n* = 1)	Cerebral ischemia (*n* = 1)			Posterior reversible encephalopathic syndrome (*n* = 1)	Proteinuria (*n* = 1), lymphopenia (*n* = 1), pneumonia (*n* = 1)			
**Other**	Two patients had a rise in LH/FSH, on subsequently missed her period					Wound healing delay (*n* = 2)			Infected port requiring removal (*n* = 2)		

HTN, hypertension * not thought to be due to drug. **HTN after 10 infusion requiring medical treatment. ***Pre-existing Grade I and II in two patients; in patient with Grade I, HTN increased to Grade II; the patient with grad II remained Grade II. ^&^Fatigue Grade I–III (*n* = 7), HTN Grade I–III (*n* = 7).

In the cases reported by Hwang et al. (2013) who were retreated with bevacizumab, there were no reported events higher than Grade II, however, they did tend to occur sooner and result in stopping treatment in three of the four patients (due to epistaxis, proteinuria, HTN, primary inflammatory arthritis, pterygoid myositis). HTN, joint pain, and proteinuria improved with increasing dosing interval. Most resolved within 1 month of stopping treatment. Two cases of HTN and proteinuria took several months to resolve. One patient had persistent grade 2 HTN 1 year after completion of therapy.

In patients with NF2 treated with bevacizumab at 5 mg/kg/dose, no thromboembolic events, hemorrhage, congestive heart failure, gastrointestinal perforation, reversible posterior leukoencephalopathy, or Grade III/IV events have been reported (Plotkin et al., 2009; Mautner et al., 2010). Grade I/II events reported were similar to those reported in patients treated at higher doses of bevacizumab.

Secondary amenorrhea was reported in a few patients. In the Phase I COG study, two of three post-menarchal females had a rise is LH and FSH, one subsequently missed her periods (Glade Bender et al., 2008). In the patient treated for renal cell carcinoma who developed amenorrhea, menses resumed 1 month after cessation of bevacizumab (Joshi and Banerjee, 2008).

The patient treated for CAT syndrome was noted to have multiple punched out boney lesions after four doses of bevacizumab (Smith et al., 2008). Bevacizumab was stopped and repeat imaging 2 months later showed complete resolution of the bony lesions.

Together, these studies indicate that bevacizumab is generally well tolerated in the pediatric population at doses up to 15 mg/kg/dose. Serious side effects seen in adults have not been reported in the pediatric population.

## Mechanisms of Resistance

Targeting VEGF pathways clearly has a role in the treatment of a broad range of human disease. Some applications, like the treatment of post-irradiation edema or CLS, relate more to VEGF-mediated regulation of vascular permeability rather than to VEGF-stimulated angiogenesis. In these circumstances bevacizumab has demonstrated significant effect and it is likely that its use as a single agent for these indications will increase. In contrast, as detailed above, in cancer treatment when the angiogenic functions are the critical target for VEGF directed therapies, bevacizumab fails in most cases to produce a lasting clinical response. Identifying the mechanisms of resistance and appropriately modifying treatment approaches will be essential for the full realization of the potential of anti-angiogenic therapies. Here we will briefly review proposed mechanisms by which cancers adapt and evade VEGF antagonism.

In discussion of and investigation into mechanisms of bevacizumab resistance, much has been made of an apparent change in the pattern of recurrence in bevacizumab-treated, versus conventionally treated, GBM. Some retrospective and prospective studies have reported an increase in the frequency of diffuse and distant recurrence after bevacizumab therapy from approximately 10–50% (Norden et al., 2008; Narayana et al., 2012a,b). However, these conclusions have been called into question by several additional studies that indicate the prevalence of pathologically identified disseminated disease at GBM recurrence in the pre-bevacizumab era is really 17% (Silbergeld et al., 1991) and the pattern of disseminated disease after bevacizumab treatment to be no different from this or from recurrence without bevacizumab treatment (Chamberlain et al., 2009; Pope et al., 2009). Preclinical models however, do support a shift in GBM growth from what has been termed an expansile to a permeative pattern to reflect its highly invasive nature (Pàez-Ribes et al., 2009; de Groot et al., 2010; Keunen et al., 2011).

The mechanisms that drive resistance to bevacizumab therapy are not yet fully defined. However, preclinical and clinical studies have revealed multiple intratumoral and systemic pathways that can contribute to tumor progression on bevacizumab therapy and these have been well reviewed by Bergers and Hanahan (2008). In response to bevacizumab-induced hypoxia, model tumors can increase expression of pro-angiogenic factors like FGFs 1 and 2 (Casanovas et al., 2005). Moreover, systemic up-regulation of FGF as well as the pro-angiogenic factor CXCL12 has also been observed in patients treated with the VEGF receptor inhibitor Cediranib (Batchelor et al., 2007). In addition, bevacizumab therapy is associated with recruitment of pro-angiogenic bone-marrow-derived cells that can directly participate in angiogenesis or provide support and protection for already formed blood vessels through the elaboration of pro-angiogenic cytokines (Song et al., 2005; Jin et al., 2006; Du et al., 2008; Shaked et al., 2009; Kioi et al., 2010). Finally, the potential for vascular mimicry and the transdifferentiation of glioma stem cells into endothelial-like cells may be an important mechanism for VEGF-independent angiogenesis (Ricci-Vitiani et al., 2010; Wang et al., 2010; Dong et al., 2011; Scully et al., 2012). It is likely that the relative contributions that each of these mechanisms makes to bevacizumab and other anti-angiogenic therapies as well as the specific circumstances in which each is most active will need to be enumerated before fully successful anti-angiogenic therapies for malignancies can be realized.

## Future Directions

In addition to developing therapeutics that can target the multiple mechanisms of angiogenesis and tumor recurrence, a great clinical challenge as we advance anti-angiogenic therapies for cancer will be the application of laboratory and imaging techniques that can better define response early in therapy. This will be especially critical in the application of anti-angiogenic therapies to brain tumors where serial biopsy is not currently available. In this regard, serum evaluation for the up-regulation of pro-angiogenic factors, especially those associated with resistance like VEGF, PIGF, PDGF, FGF, IL8, CXCL12 may provide insight into shifts in response (Murukesh et al., 2010). Recent clinical trial data with several anti-angiogenic tyrosine kinase inhibitors have demonstrated that increased VEGFA and PIGF levels are associated with treatment and reduced survival (Drevs et al., 2005; Rini et al., 2008; Bass et al., 2010; Nikolinakos et al., 2010).

In addition, imaging, particularly MRI-based spectroscopy, perfusion, diffusion, and oxygen consumption sequences may be able to detect functional changes in the vasculature relevant to anti-angiogenic responses and recurrent angiogenesis (Pope et al., 2009, 2011; Ellingson et al., 2011; Ben Bashat et al., 2012). As examples, Pope et al. (2012) recently published that baseline ADC histograms are predictive of 6-month survival in patients with recurrent GBM treated with bevacizumab and Sorensen et al. (2012) reported that increased perfusion in recurrent GBM patients treated with cediranib is also predictive of survival. In addition, acute metabolic changes in response to cediranib therapy are detectable with MRI-spectroscopy patients and should be evaluated for their correlation with survival (Kim et al., 2011).

Finally, PET imaging for therapy-induced changes in expression of specific components of angiogenic pathways as well as for changes in vascular function is an additional promising approach for detecting response to anti-angiogenic therapies that is just entering clinical evaluation (Chen and Chen, 2011; Schwarzenberg et al., 2012). Schwarzenberg et al. (2012) have reported exciting new findings demonstrating the superiority of 3′-deoxy-3′-18F fluorothymidine imaging to conventional MRI imaging for detecting early response to bevacizumab therapy in patients with recurrent GBM.

Together, sophisticated and combinatorial monitoring of response to anti-angiogenic therapies may support the timing of adjunctive interventions to circumvent resistance mechanisms and enhance efficacy.

## Conflict of Interest Statement

The authors declare that the research was conducted in the absence of any commercial or financial relationships that could be construed as a potential conflict of interest.
